# Evaluation of tilapia skin (*Oreochromis niloticus*) frozen, frog skin (*Rana catesbeiana*), and tilapia skin (*Oreochromis niloticus*) sterilized with gamma rays dressings, in equine skin wound healing

**DOI:** 10.3389/fphar.2026.1896365

**Published:** 2026-09-08

**Authors:** Rachel Pereira da Rocha Miranda, Beatriz Alves de Miranda, Andressa Borges Aranha, Maria Fernanda Bruno Gomes Ramos, Edson Pereira De Oliveira, Bruno Stéfano Lima Dallago, José Adorno, Isabel Luana De Macêdo, Márcio Botelho De Castro, Rita De Cássia Campebell

**Affiliations:** 1 Hospital Escola de Grandes Animais, Faculdade de Agronomia e Medicina Veterinária (FAV), Universidade de Brasília (UnB), Brasília, Brazil; 2 Burn Unit of Asa Norte Regional Hospital (HRAN), Brasília, Brazil

**Keywords:** frog skin, horse, thermography, tilapia skin, wounds

## Abstract

**Background:**

Skin wounds are common in horses due to species-specific behavioral responses. Equine skin morphology, vascularization, and inflammatory responses to wounds are comparable to those in humans, supporting the translational relevance of equine wound-healing studies. Investigating biomaterials such as tilapia and frog skin may improve wound management. This study demonstrated that sterilized tilapia and frog skin exhibit promising wound-healing properties.

**Materials and Methods:**

Macroscopic, microscopic, and thermographic aspects of second-intention wound healing were compared in six horses with surgically induced skin wounds treated topically with frozen tilapia skin (FTS), sterilized tilapia skin (STS), sterilized frog skin (SFS), or lactated Ringer’s solution (CW). Wounds were created bilaterally in the thoracolumbar region, with one side designated for histopathological evaluation and the contralateral side for macroscopic assessment, including photographic documentation, wound area measurement, contraction rate, and thermographic analysis. Treatments were applied daily, and wound progression was monitored for 28 days. Evaluations were performed on days 0, 3, 7, 14, 21, and 28 after wound induction.

**Results:**

No clinical abnormalities were observed in the horses throughout the study. Regarding wound area, the FTS group exhibited the largest lesions on day 14 compared with the CW group. Over time, the CW and FTS groups showed reduced wound contraction, with increased wound area on day 7. Histopathological evaluation revealed a significant increase in polymorphonuclear cells (PMNs) in all groups on day 3, which persisted until day 7 in the CW group and until day 28 in the FTS group. Thermographic analysis showed no significant differences among treatments.

**Conclusion:**

Sterilized tilapia skin reduced the inflammatory response, enhanced wound-edge contraction, and accelerated healing. Frog skin was associated with faster reduction of edema and hemorrhage, as well as more pronounced fibroplasia during the early stages of healing. In contrast, frozen tilapia skin showed limitations, including lower adherence to the wound bed, increased contamination, and delayed healing.

## 1 Introduction

Skin wounds in horses represent a major global health and welfare concern and are among the leading causes of morbidity, mortality, and euthanasia in the species (Sole et al., 2015; Theoret, 2017). Wound healing is a complex process involving overlapping phases of inflammation, proliferation, and remodeling. In horses, healing is generally less efficient than in ponies because the inflammatory phase is milder yet prolonged, increasing susceptibility to infection and extending the proliferative phase, often resulting in chronic or nonhealing wounds. Healing also varies by anatomical location, with body wounds healing faster than distal limb lesions, which are more prone to contamination, excessive movement, limited soft-tissue coverage, and exuberant granulation tissue (EGT) formation (Maher and Kuebelbeck, 2018; Provost, 2019; Anantama et al., 2022).

Equine skin morphology, vascularization, and inflammatory responses closely resemble those of humans, making horses a valuable translational model for wound-healing studies. Compared with smaller experimental models, horses offer practical advantages, including a larger skin surface area, wounds more comparable to human lesions, and the feasibility of long-term monitoring without the need for euthanasia (Harman et al., 2021; Sparks et al., 2021).

Tilapia skin, particularly from *Oreochromis niloticus*, has been increasingly studied as a wound-healing biomaterial in veterinary and human medicine. Rich in type I collagen, it exhibits high tensile strength, flexibility, and biocompatibility. Its biological properties include maintaining wound hydration, protecting against contamination, promoting a moist healing environment, reducing exuberant granulation tissue, and exerting antimicrobial activity. Structurally, tilapia skin resembles human skin and demonstrates high elasticity and resistance. Its use as an occlusive biological dressing for superficial second-degree burns in humans has been associated with pain reduction and lower treatment costs (Costa et al., 2020; Lima Junior et al., 2020). Studies at the Institute for Energy and Nuclear Research (IPEN) demonstrated that gamma irradiation sterilization does not alter the histological structure of tilapia skin (Alves et al., 2015; Alves et al., 2018; Costa et al., 2020). Additionally, studies using fresh tilapia skin in equine wounds reported improved tissue repair and reduced exuberant granulation tissue formation without adverse effects (Silva et al., 2019; Silva, 2022).

Frog skin, particularly from *Rana catesbeiana*, has shown promise as a wound-healing biomaterial because of its high collagen content, flexibility, biocompatibility, and natural antimicrobial properties (Bazaz et al., 2013; Mashreghi et al., 2013). Additionally, frog skin has been linked to increased proliferation of epidermal and dermal cells, which are essential for tissue repair (Raghavan et al., 2010; Li et al., 2023). Recently, the amphibian-derived peptide OA-RD17 demonstrated beneficial therapeutic effects in burn and cutaneous wound models (Li et al., 2023).

The present study evaluated clinical findings, wound area, contraction rate, histopathological changes, and thermographic parameters in experimentally induced equine wounds treated with frozen tilapia skin, sterilized tilapia skin, and frog skin. Healing time and tissue repair quality were compared across treatment groups using standardized thoracolumbar wounds in six horses.

## 2 Materials and methods

### 2.1 Animals

This study was approved by the Ethics Committee on Animal Use of the University of Brasília (CEUA/UnB; SEI No. 23106.027371/2022-70) on 26 May 2022, in accordance with national ethical regulations. The horses used in the experiment had been abandoned in public areas and were rescued by the Department of Agriculture of the Federal District, Brazil. Before inclusion, all animals received clinical care, vaccination, deworming, and appropriate nutritional management until they were deemed healthy for the study. After the experimental period, all horses were allowed 35–40 days for complete wound healing and were subsequently adopted by new owners.

Six clinically healthy horses were selected, including one female and five geldings, all mixed-breed, with a mean body weight of 277.67 kg. Clinical examination, hematologic testing, and serum biochemistry (urea, creatinine, gamma-glutamyl transferase, aspartate aminotransferase, and albumin) showed no abnormalities. Deworming was performed orally with ivermectin and praziquantel (Padock Plus NF, CEVA, Paulínia, SP, Brazil), and all horses received prophylactic antitetanus serum (Venco-sat, Vencofarma, Londrina, PR, Brazil). Animals were housed individually in stalls under appropriate hygienic conditions and fed commercial concentrate (Nutrina Equinos Premium, Nutrina Rações e Minerais, Brasília, DF, Brazil), coast-cross hay, and water *ad libitum*. Heart rate, respiratory rate, mucous membrane color, capillary refill time, rectal temperature, intestinal motility, attitude, and appetite were monitored daily throughout the study.

### 2.2 Preparation of frozen tilapia skin (FTS)

Nile tilapia (*O. niloticus*) skins were obtained from local fish suppliers in Brasília, DF, Brazil. Fish weighing approximately 800–1,000 g were euthanized by thermal shock, followed by exsanguination. Skins were removed, cleaned, and processed following Alves et al. (2015) and Lima Verde et al. (2021). Residual muscle tissue was removed with scalpels and forceps (Supplementary Figure 1A), while scales were left intact. Skins were immersed in sterile 0.9% saline (Supplementary Figure 1B), individually packaged in sterile plastic envelopes, and stored frozen at −20 °C until use (Supplementary Figure 1C).

### 2.3 Preparation of sterilized tilapia skin (STS) and sterilized frog skin (SFS)

Tilapia skin preparation followed the same initial procedures described for FTS. Frog skins were obtained from Ranário Laranjeiras–Ranajax (Anápolis, GO, Brazil), a certified commercial frog farm operating under the Brazilian Code of Responsible Conduct for Frog Farming. Skins were collected from healthy frogs slaughtered under Federal Inspection Service supervision. After removal and cleaning (Supplementary Figure 1D), skins were divided into 1-kg sublots and refrigerated at 2 °C.

Both frog and tilapia skins were dehydrated on metal plates coated with fish or frog oil to maintain hydration (Supplementary Figure 1E). In a controlled environment, the plates were heated to 28 °C for 45 min to allow the skins to detach (Supplementary Figure 1F). After this phase, the skins were cooled and then packaged in surgical-grade paper, sealed, and sent for sterilization with Co-60 gamma rays (gamma energy of approximately 1.25 MeV) in a Gammacell GC220 MDS Nordion irradiator (Canada) at a dose rate of approximately 1.44 kGy/h, with a total dose of 25 kGy (Supplementary Figure 1G). Sterilization took place at the Gamma Radiation Laboratory (GAMALAB) of the Nuclear Energy Department of the Federal University of Pernambuco–Recife/PE.

### 2.4 Wound preparation

Prior to surgery, horses were fasted for 12 h without food and 6 h without water. Sedation was induced with 10% xylazine hydrochloride (Equisedan®, J.A. Saúde Animal, Patrocínio Paulista/SP, Brazil) administered intravenously at 1 mg/kg. Trichotomy was performed bilaterally in the thoracolumbar region, cranial to the tuber coxae and ventral to the transverse processes of the lumbar vertebrae (L1–L6). Surgical antisepsis was performed with povidone-iodine scrub followed by 70% alcohol (Supplementary Figure 2A).

Immediately thereafter, wound templates were outlined with a violet surgical marker and measured with a sterile ruler (Supplementary Figure 2B). Local infiltrative anesthesia was administered with 2% lidocaine without vasoconstrictor (Lidocaine Hydrochloride 2%®, Hipolabor Farmacêutica, Belo Horizonte/MG, Brazil). On both sides of the thoracolumbar region, cranial to the sacrum and ventral to the lumbar transverse processes, four square, full-thickness skin wounds (3 × 3 cm) spaced 5 cm apart were surgically created using a sterilizable quadrangular template. Incisions included the skin and subcutaneous tissue, exposing the underlying muscle fascia (Supplementary Figures 2C,D).

### 2.5 Microbiological evaluation of frog and tilapia skins

The microbiological evaluation of the biomaterials was adapted from the methodology described by Lima Júnior et al. (2016), (2017). Samples of frozen tilapia skin (FTS), sterilized tilapia skin (STS), and sterilized frog skin (SFS) were prepared according to the clinical application protocol and washed with sterile 0.9% saline solution for 5 minutes under gentle agitation to remove surface residues.

Skin fragments were then macerated and homogenized in 1 mL of sterile saline to obtain a slightly turbid suspension. Aliquots of 0.1 mL were inoculated in duplicate onto Blood agar, MacConkey agar, and Sabouraud agar plates. Cultures were incubated at 37 °C for 72 h, then macroscopically evaluated for microbial growth.

### 2.6 Treatments

Horses were randomly assigned to macroscopic and microscopic evaluations. In three animals, the right thoracolumbar region was designated for clinical assessment and the left for biopsy collection; in the remaining three animals, the opposite arrangement was used. Treatment allocation was randomized using a die to minimize selection bias. Predefined die numbers corresponded to the left and right sides, and treatment allocation was determined by the outcome of each roll. The same procedure was used to define the distribution sequence of the experimental groups.

Control wounds (CW) were treated with Ringer’s lactate solution and covered with two overlapping sterile gauze pads secured with adhesive tape. Before application, frozen tilapia skin was thawed in Ringer’s lactate solution containing 0.2% chlorhexidine for 20 min, as described by Silva et al. (2019), then rinsed with Ringer’s lactate solution, dried with a sterile compress, trimmed to wound size, and secured with gauze, adhesive tape, and bandage material.

For STS and SFS applications, sterile packages were opened aseptically. The biomaterials were cut to fit the wounds, immersed in Ringer’s lactate solution for 2 min, dried on sterile compresses, and applied directly to the wound bed, followed by bandaging with gauze and adhesive tape (Supplementary Figure 2E). In the FTS, STS, and SFS groups, dressings were changed daily until day 7 (D7) and thereafter only if biomaterial detachment or excessive exudation occurred. All wounds were cleansed with Ringer’s lactate solution. In the CW group, dressings were replaced daily throughout the 28-day experimental period.

All horses received phenylbutazone (4.4 mg/kg × kg−1; Equipalazone®, CEVA, Paulínia/SP, Brazil) and dipyrone (25 mg/kg × kg−1 IV, SID) immediately after surgery and for three consecutive days to manage pain and edema.

### 2.7 Clinical and photographic evaluation of the wounds

Clinical and photographic evaluations were performed on the day of surgery (D0) and on postoperative days 3 (D3), 7 (D7), 14 (D14), 21 (D21), and 28 (D28). Clinical assessment included evaluation of edema, hyperemia, local hemorrhage, crust formation, clot presence, exudate type and quantity, granulation tissue formation, and epithelialization.

Standardized photographic documentation, including a ruler for calibration, was used to objectively measure wound area and calculate contraction rates. Wound measurements were analyzed with ImageJ2 software (Wayne Rasband, National Institutes of Health, Bethesda, MD, United States; http://rsb.info.nih.gov/ij/download.html; accessed 18 September 2024).

Wound contraction was calculated according to Ramsey et al. (1995): contraction rate (%) = 100 × (W0 − WA)/W0, where W0 is the original wound area immediately after wound creation and WA is the wound area at the evaluation time (0, 3, 7, 14, 21, or 28 days), both expressed as percentages.

Data on wound area and contraction rate were analyzed using a split-plot design with repeated measures over time, followed by analysis of variance (ANOVA) and Tukey’s *post hoc* test. Normality and homogeneity of variances were assessed, and significance was set at *p* < 0.05.

### 2.8 Biopsy and histopathological evaluation

Skin biopsies were collected on D0, D3, D7, D14, D21, and D28 using a 6-mm punch under local infiltrative anesthesia with 1 mL of 2% lidocaine without vasoconstrictor (Lidocaine Hydrochloride 2%®, Hipolabor Farmacêutica, Belo Horizonte/MG, Brazil). Samples were obtained in a central, clockwise sequence, avoiding previously biopsied regions.

Histopathological analyses were conducted at the Veterinary Pathology Laboratory of the University of Brasília using light microscopy, photomicrography, and semiquantitative assessment. Tissue fragments were fixed in 10% buffered formalin (pH 7.0). Samples were routinely embedded in paraffin, sectioned at 4 μm, stained with hematoxylin and eosin (H&E), and examined under light microscopy.

Semiquantitative histopathological evaluation assessed inflammatory parameters (neutrophils, histiocytes, eosinophils, and monocytes), proliferative changes (fibroblasts and neovascularization), and the presence of ulcers and hemorrhage. Lesions were graded as absent/rare (−), mild (+), moderate (++), or marked (+++). Median histopathological scores were analyzed using the Kruskal–Wallis test. Statistical analyses were performed using SAS® software version 9.4 (Cary, NC, United States), with significance set at 5%.

### 2.9 Thermographic evaluation

Thermographic assessments were performed at D0, D3, D7, D14, D21, and D28 during clinical examinations to detect local temperature variations associated with inflammation. These findings were correlated with laboratory and histopathological results. Images were obtained using a FLIR® T420 thermographic camera and analyzed with FlirTools® software. The equipment had a resolution of 320 × 240 pixels, a thermal sensitivity of 0.04 °C, and a temperature range of −20 °C to 650 °C. Temperature measurements were obtained from three predefined regions: the wound bed, wound edges, and periwound area (Supplementary Figures 2F–H respectively), using the ellipse and box measurement tools in FlirTools® software (v5.13, FLIR Systems). Three images were recorded for each region, and the sharpest image was selected for analysis.

To ensure image reliability, precautions included minimal restraint, control of environmental radiation, monitoring ambient temperature, and removal of artifacts such as dust, mud, sweat, or sawdust. Horses were restrained in a shaded, draft-free pen (Turner, 2001). Thermographic examinations were standardized and conducted before 10:00 a.m., with ambient temperature maintained at approximately 20 °C.

Before wound induction, the thoracolumbar region was brushed for 10 min to dissipate transient heat generated by manipulation, as recommended by Soroko and Howell (2018). Images were then obtained at a standardized distance of 1 m and at a perpendicular angle (90°) to the wound surface. During postoperative evaluations, dressings were removed 10 min before image acquisition, and nonadherent debris was gently removed without inducing bleeding.

## 3 Results

### 3.1 Physical assessment of horses and wounds

Throughout the experimental period, no clinically significant changes related to the surgical wounds were observed in any horse. During wound creation and biopsy procedures, cardiorespiratory parameters remained stable, indicating adequate anesthetic blockade and satisfactory procedural tolerance.

From the first to the seventh postoperative day, all wounds exhibited edema and increased sensitivity, and the animals became more reactive during dressing changes, although all physiological parameters remained within normal ranges for the species. During biopsy collection, no pain responses were observed; however, increased sensitivity to palpation of the wound margins was noted during the first 7 days, despite postoperative phenylbutazone administration.

Macroscopic evaluation revealed hemorrhage, clot formation, exudation, and crust formation in all groups during the first week, with these changes more pronounced in the frozen tilapia skin (FTS) group. The acute inflammatory phase persisted until day 7 (D7) and was characterized by wound enlargement, edema, heat, tenderness, exudate, and granulation tissue formation. Granulation tissue was observed in all groups by D7, whereas epithelialization became evident from D14 onward, coinciding with progressive wound contraction, although this process was delayed in the FTS group. By D28, wounds showed more than 80% healing, with extensive reepithelialization.

### 3.2 Wound area and wound contraction rate

**FIGURE 1:**
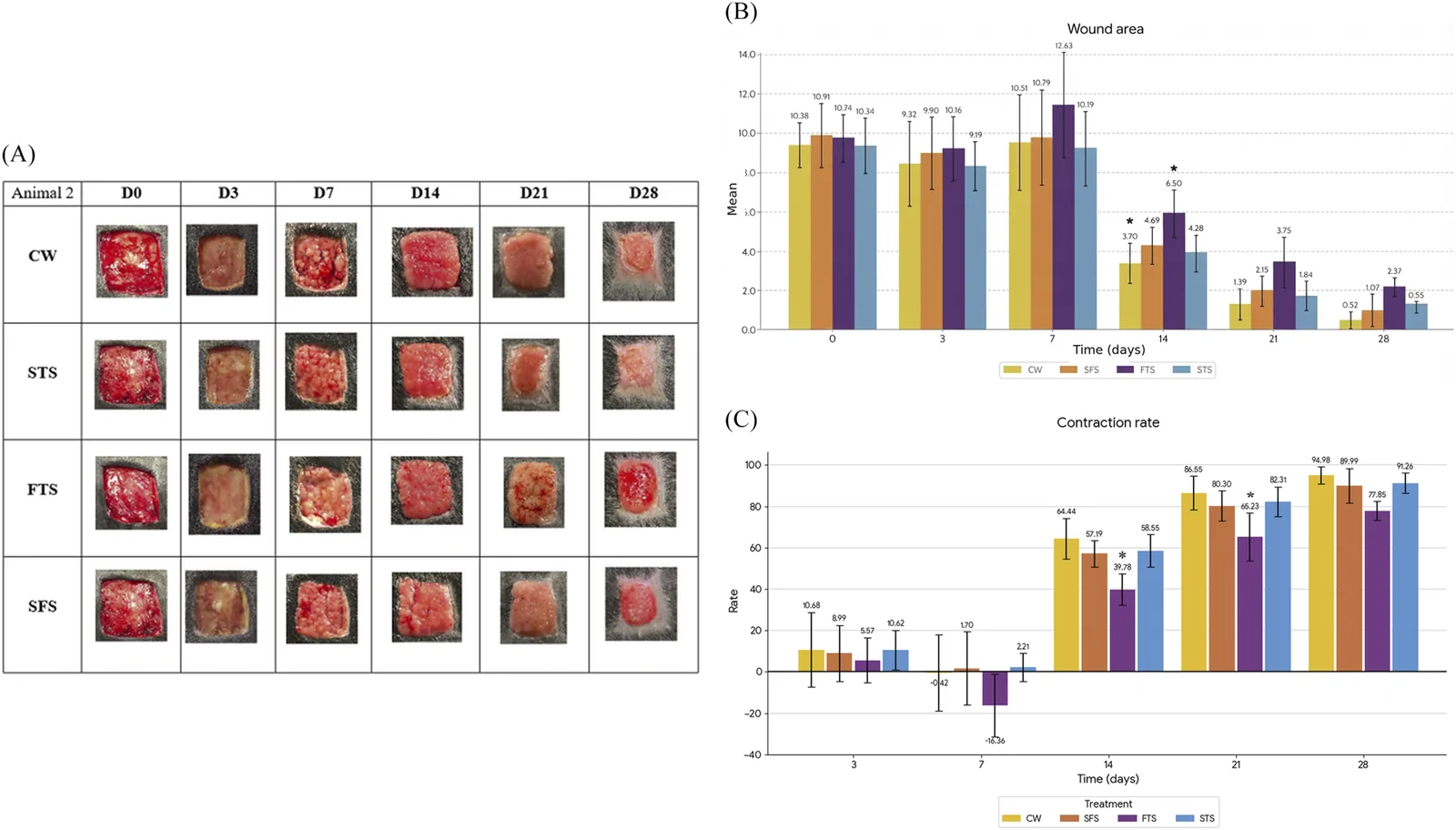
**(A)** Photographic images of the wounds of the control wound (CW), frozen tilapia skin (FTS), sterilized tilapia skin (STS) and sterilized frog skin group (SFS), on days 0 (D0), 03 (D3), 07 (D7), 14 (D14), 21 (D21), 28 (D28), in Animal 2. **(B)** Mean of wound area (cm^2^) and **(C)** mean of contraction rate (%) for the control group (CW), frozen tilapia skin (FTS), sterilized tilapia skin (STS) and sterilized frog skin group (SFS), on days 0 (D0), 03 (D3), 07 (D7), 14 (D14), 21 (D21), 28 (D28).

A non-significant increase in wound area was observed on D7 in the control wound (CW) and FTS groups. On D14, the FTS group had a significantly larger wound area than the CW group. Over time, all groups showed a significant reduction in wound area relative to D0, beginning on D14 (Figures 1A,B).

Negative contraction rates were observed on D7 in the CW and FTS groups, reflecting transient wound enlargement during the acute inflammatory phase. From D14 onward, all groups showed significant increases in wound contraction rates. On D14 and D21, the FTS group had significantly lower contraction rates than the CW group. Although differences were not statistically significant thereafter, the CW group had the highest contraction rates, followed by sterilized tilapia skin (STS), sterilized frog skin (SFS), and FTS (Figures 1A,C).

### 3.3 Microbiological culture

Gamma irradiation sterilization was fully effective for both tilapia and frog skins, with no bacterial or fungal growth detected in the STS and SFS groups. In contrast, frozen tilapia skin samples stored at −20 °C exhibited substantial microbial growth in all culture media tested. Morpho-tinctorial analysis identified Gram-positive cocci as well as Gram-positive and Gram-negative bacilli. Data not shown.

### 3.4 Histopathological evaluation

#### 3.4.1 Polymorphonuclear cells

**FIGURE 2:**
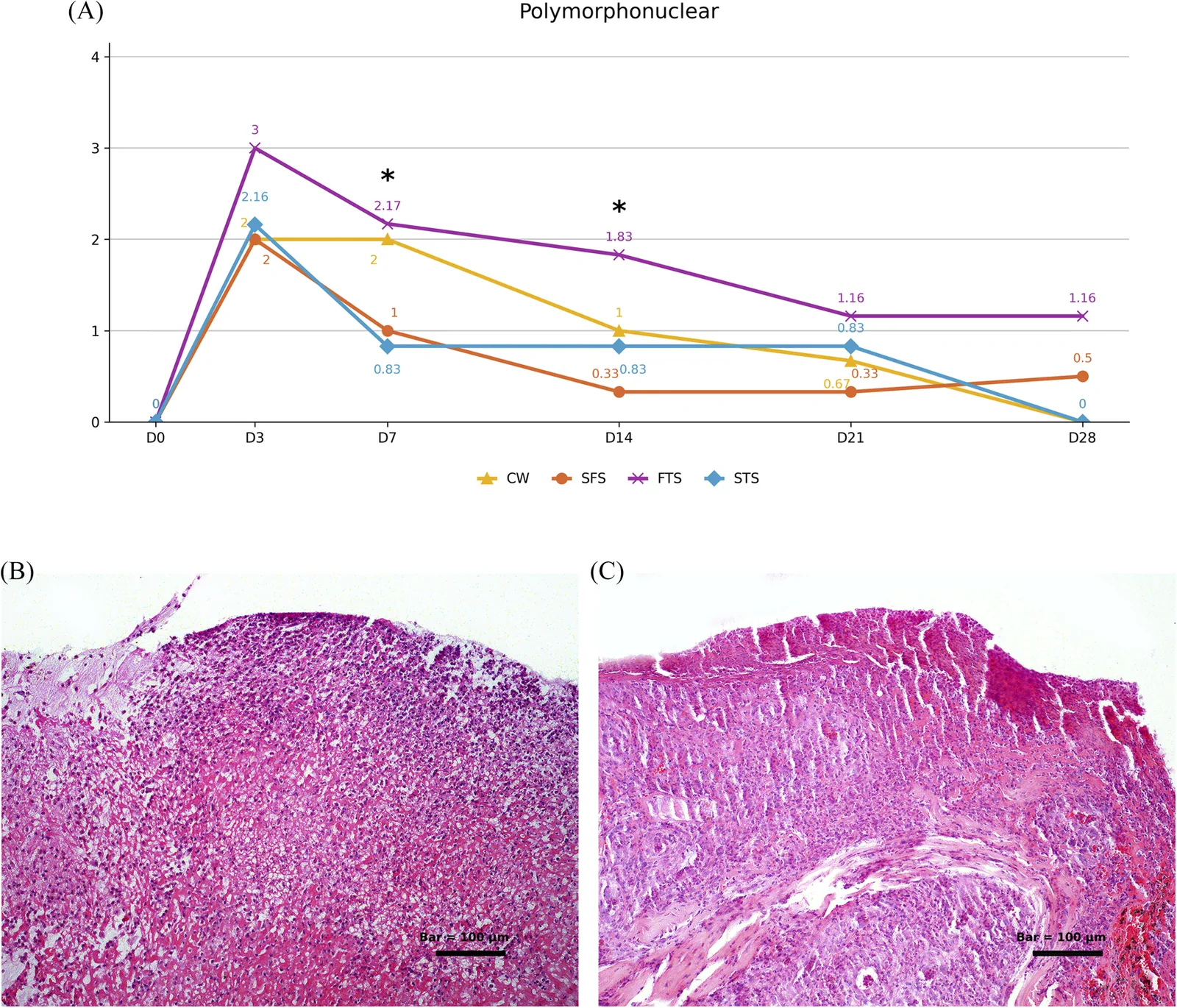
**(A)** Mean polymorphonuclear leukocytes of the control wound (CW), frozen tilapia skin (FTS), sterilized tilapia skin (STS) and sterilized frog skin group (SFS), on days 0 (D0), 03 (D3), 07 (D7), 14 (D14), 21 (D21), 28 (D28). Histological image of the **(B)** control wound (CW) and the **(C)** frozen tilapia skin (FTS) on D7, with intense polymorphonuclear inflammation with cellular debris (black circle), hemorrhage (*) and edema (red arrows). HE, 10x objective. Animal 4.

A significant increase in polymorphonuclear cells (PMNs) was observed in all groups on D3. Elevated PMN levels persisted until D7 in the CW group and until D28 in the FTS group. Compared with the other treatments, the FTS group had significantly higher PMN counts on D3 and D28, and higher values than the SFS and STS groups on D7 and D14 (Figure 2).

#### 3.4.2 Histiocytes assessment

**FIGURE 3:**
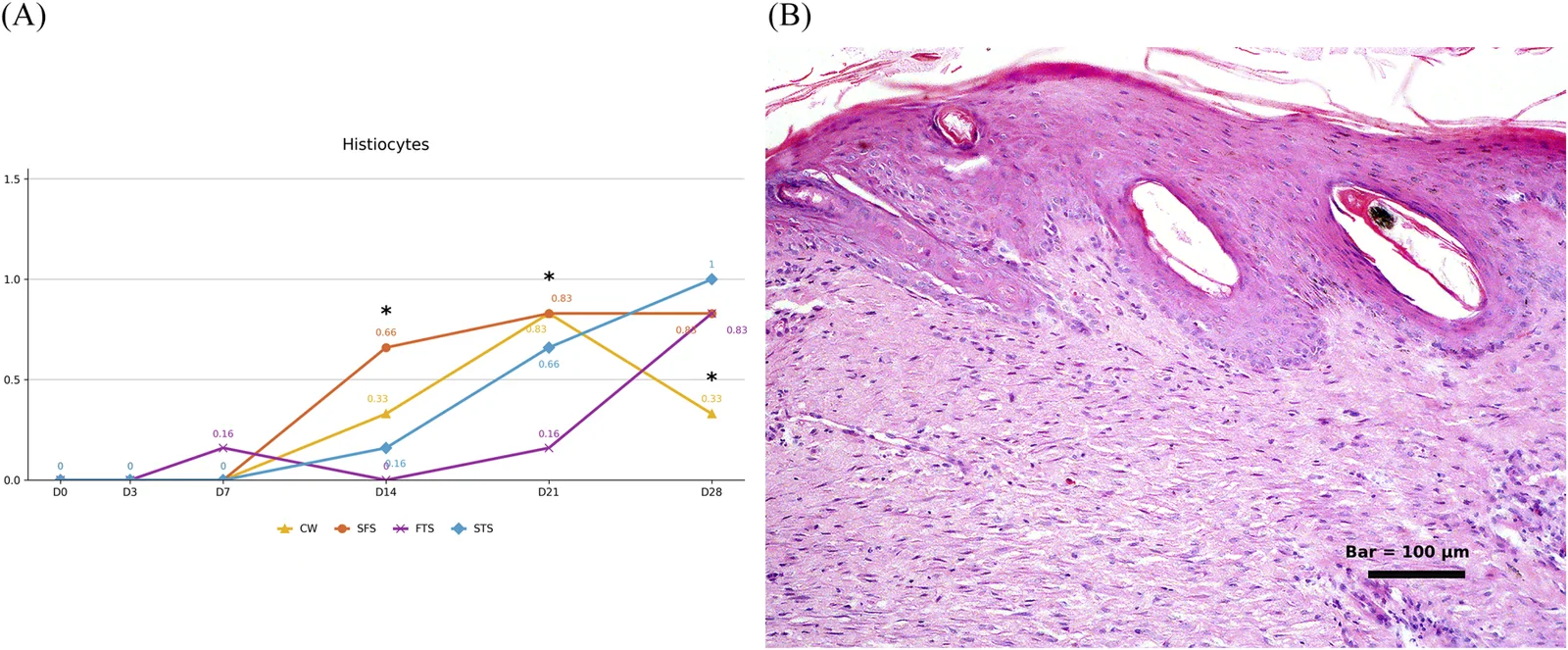
**(A)** Mean histiocytes of the control wound (CW), frozen tilapia skin (FTS), sterilized tilapia skin (STS) and sterilized frog skin group (SFS), on days 0 (D0), 03 (D3), 07 (D7), 14 (D14), 21 (D21), 28 (D28). **(B)** Histological image of the wound from the sterilized tilapia skin (STS) on day 28, with intense fibroplasia, neovascularization, and discrete mononuclear inflammatory infiltrate (circle). HE, 10x objective. Animal 4.

Histiocytic infiltration increased significantly from D14 onward in the SFS group, from D21 in the STS and CW groups, and on D28 in the FTS group. Significant differences were observed among treatments. The SFS group had the highest histiocyte counts on D14, whereas the lowest counts were observed in the FTS and CW groups on D21 and D28, respectively (Figure 3).

#### 3.4.3 Edema

**FIGURE 4:**
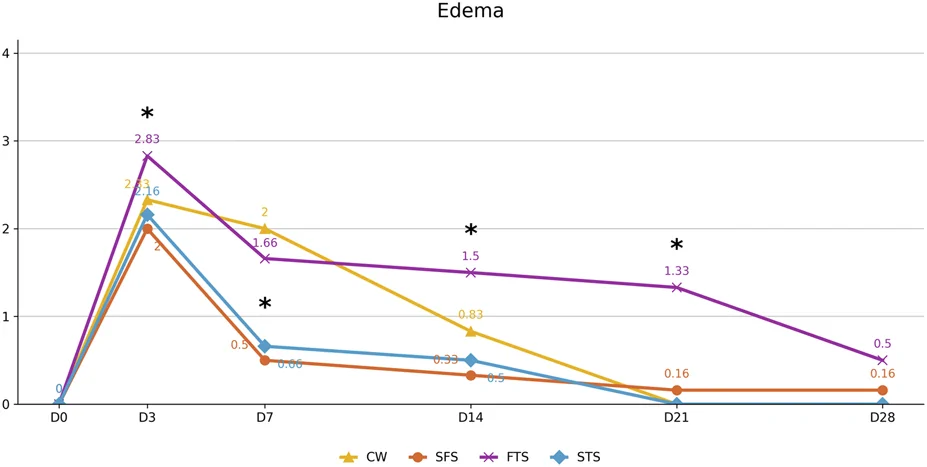
Mean edema of the control wound (CW), frozen tilapia skin (FTS), sterilized tilapia skin (STS) and sterilized frog skin group (SFS), on days 0 (D0), 03 (D3), 07 (D7), 14 (D14), 21 (D21), 28 (D28).

Edema increased significantly across all groups on D3 and remained elevated until D14 in the CW group and until D21 in the FTS group. On D3, the SFS group had lower edema scores than FTS, and on D7, both the SFS and STS groups had lower scores than CW and FTS. Conversely, the FTS group exhibited significantly greater edema on D14 than SFS and STS, and on D21 than all other groups (Figures 1A, 4).

#### 3.4.4 Hemorrhage and neovascularization assessment

**FIGURE 5:**
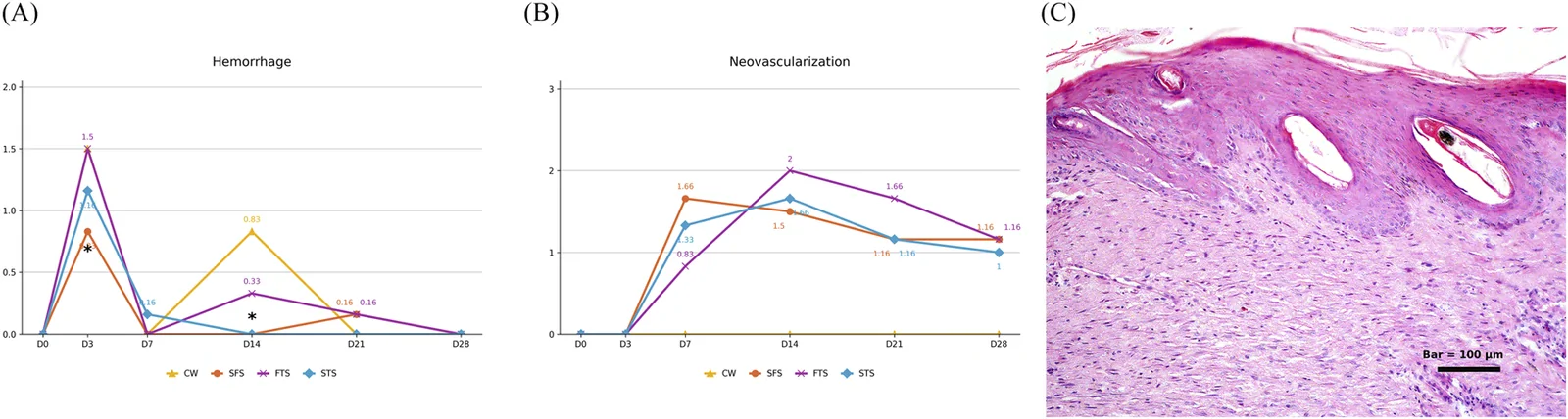
**(A)** Mean of wound hemorrhage assessment and **(B)** mean of neovascularization of the control wound (CW), sterilized frog skin group (SFS), frozen tilapia skin group (FTS) and sterilized tilapia skin group (STS), on days 0 (D0), 03 (D3), 07 (D7), 14 (D14), 21 (D21), 28 (D28). **(C)** Histological image of the wound from the sterilized tilapia skin (STS) on day 28, with intense fibroplasia, neovascularization, and discrete mononuclear inflammatory infiltrate (circle). HE, 10x objective. Animal 4.

Hemorrhage was predominantly observed during the first 7 days in all groups, persisting through D14 in the CW and FTS groups. On D3, hemorrhage scores were significantly lower in the SFS group than in the CW and FTS groups, and on D14, scores were lower in the SFS and STS groups (Figures 5A,C).

Neovascularization increased significantly in all groups from D7 onward, consistent with progression into the proliferative phase of healing (Figures 5B,C).

#### 3.4.5 Fibroplasia assessment

**FIGURE 6:**
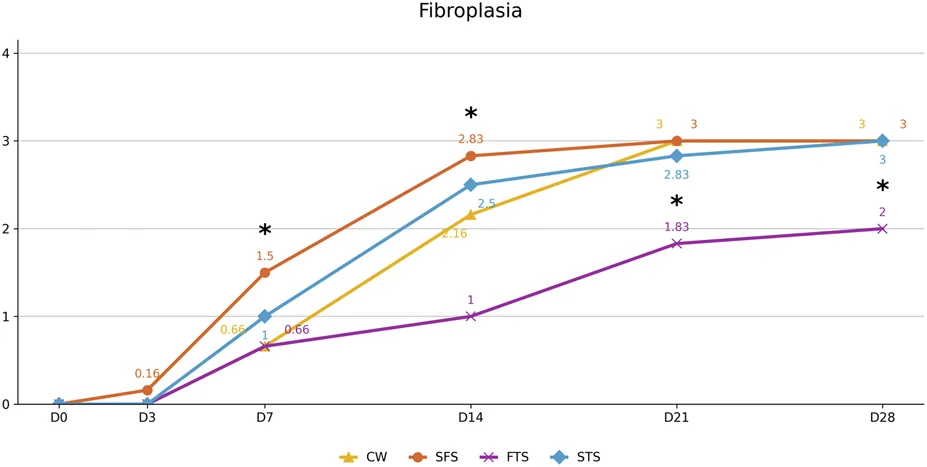
Mean fibroplasia of the control wound (CW), frozen tilapia skin (FTS), sterilized tilapia skin (STS) and sterilized frog skin group (SFS), on days 0 (D0), 03 (D3), 07 (D7), 14 (D14), 21 (D21), 28 (D28).

During the proliferative phase, fibroplasia increased significantly in all groups from D7 onward. The SFS group exhibited higher fibroplasia scores on D7 and D14 than the CW and FTS groups, whereas the FTS group showed the lowest fibroplasia indices from D14 to D28. By D28, wounds treated with FTS still showed persistent inflammation, marked by marked PMN infiltration, mild fibroplasia, neovascularization, and residual hemorrhage (Figure 6).

### 3.5 Thermographic assessment

**FIGURE 7:**
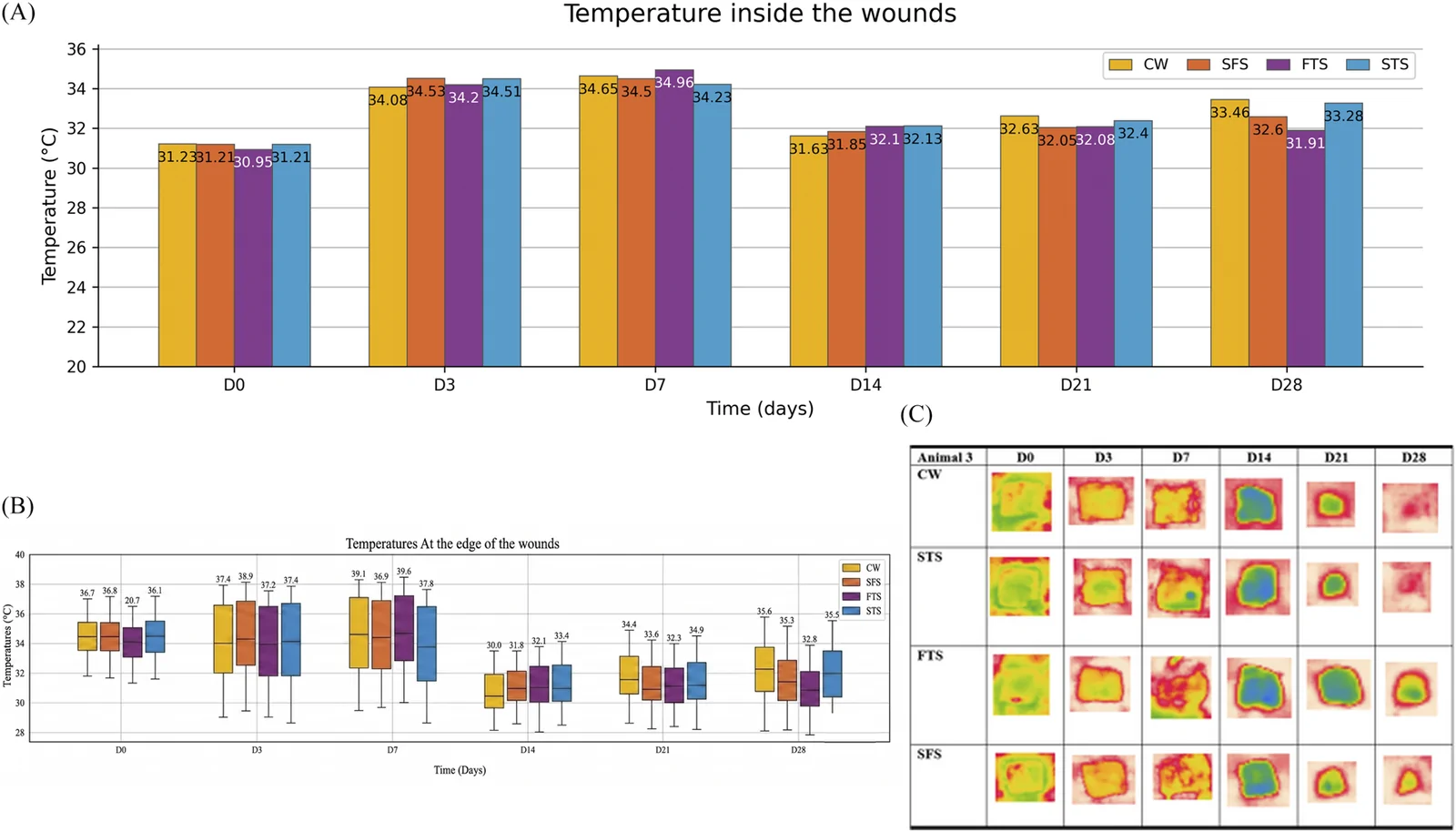
Temperature measured with a thermograph, **(A)** inside **(B)** at the edge and **(C)** inside the wounds of the control wound (CW), frozen tilapia skin (FTS), sterilized tilapia skin (STS) and sterilized frog skin group (SFS), on days 0 (D0), 03 (D3), 07 (D7), 14 (D14), 21 (D21), 28 (D28).

Within the wound bed, no significant differences were observed among treatment groups. However, all groups demonstrated a significant increase in local temperature from D3 to D7, which persisted until D14 in the FTS group and until D28 in the STS group (Figures 7A,C).

At the wound edges, no significant intergroup differences were detected, although all groups exhibited increased temperatures between D3 and D7 (Figure 7B). In the periwound region, no significant differences were observed among groups. A mild, non-significant temperature increase occurred on D3, followed by a progressive reduction from D7 onward.

## 4 Discussion

Given the close anatomical and physiological similarities between equine and human skin, the horse is a valuable translational model for studying cutaneous wound healing and evaluating novel therapeutic strategies (Harman et al., 2021). Equine and human skin share a comparable architectural organization, but humans have sparse hair follicles, whereas horses have a dense follicular distribution (Talukdar et al., 1972; Summerfield et al., 2015). In the present study, the standardized experimental wound model enabled direct comparison of treatment responses while minimizing variation due to wound size, location, and management. Consequently, the observed differences in healing progression are more likely to reflect treatment-related effects than methodological variability.

Clinical wounds may be highly heterogeneous, and their healing patterns and rates are directly influenced by factors related to the lesion, the patient, the stage of tissue repair, microbial burden, and wound chronicity (Bjarnsholt et al., 2008; Wilmink et al., 2020). This study attempted to minimize bias by standardizing wound dimensions (Madison and Gronwall, 1992), anatomical location, postoperative management, and treatment allocation, thereby reducing potential confounding factors and strengthening the interpretation that the observed outcomes resulted primarily from the therapeutic interventions. The methodology used herein was comparable to that of previous equine wound-healing studies (Martins et al., 2003; Souza et al., 2006; Oliveira et al., 2012; Lucas et al., 2017; Resende et al., 2024; Campebell et al., 2025).

Although the study included only six animals, this sample size is consistent with previous experimental wound-healing studies (Martins et al., 2003; Souza et al., 2006; Oliveira et al., 2012; Celeste et al., 2013; Ferreira et al., 2019; Kauer et al., 2020). Some studies have used more animals (Harmon et al., 2017; Lucas et al., 2017), but the within-animal experimental design, in which each horse served as its own control, may reduced biological variability and increased statistical efficiency while adhering to the principles of animal reduction in experimental research.

The experimental design, with the division of wounds into opposite sides, minimized procedural bias by separating wounds used for macroscopic assessment from those designated for biopsy collection, preventing tissue sampling from affecting measurements of wound contraction and area (Martins et al., 2003; Souza et al., 2006). Likewise, random allocation of treatments across anatomical sites further reduced allocation bias (Kauer et al., 2020). Together, these methodological approaches increased the robustness and internal validity of the comparisons among treatment groups.

In this study, sedation combined with local infiltrative anesthesia was used to ensure animal comfort during wound induction, consistent with previous studies (Souza et al., 2006; Lucas et al., 2017). In addition, the administration of phenylbutazone during the first three postoperative days provided analgesia and improved horse welfare, despite mild discomfort observed during dressing changes. Similar protocols have been reported in related investigations (Oliveira et al., 2012; Martins et al., 2013; Tracey et al., 2014; Lucas et al., 2017; Resende et al., 2024; Campebell et al., 2025). Discomfort during dressing changes in the first days after wound creation has been reported (Gomez et al., 2004; Resende et al., 2024), as observed in the horses with experimentally induced wounds, and satisfactorily controlled with the instituted therapeutic protocol.

The present study demonstrated that the biological origin and processing of xenograft biomaterials substantially influenced wound healing outcomes in horses. Although both tilapia and frog skin have been proposed as promising biological dressings because of their collagen-rich extracellular matrices and bioactive components (Gomes et al., 2007; Alves et al., 2015), their performance varied depending on the processing method used prior to application.

Tilapia skin consists predominantly of organized type I collagen fibers arranged in parallel and transverse orientations, conferring high tensile strength and elasticity. Because collagen is biocompatible and biodegradable, it is a major component of wound biomaterials (Alves et al., 2015). Tilapia is also among the most widely consumed fish worldwide, yet only a small proportion of its skin is reused, making it a low-cost, sustainable biomaterial (Alves et al., 2015). Neither frozen nor sterilized tilapia skin likely induced allergic reactions or signs of irritation in the animals, as previously observed (Costa et al., 2020).

Frog skin is one of the earliest biological xenografts used for wound coverage and has regained interest in regenerative medicine, particularly through products derived from *R. catesbeiana* (Sai et al., 1995; Sarto Piccolo et al., 2008). Its therapeutic potential is attributed to structural proteins, lipids, antimicrobial peptides, and bioactive dermal secretions that promote antimicrobial activity and tissue repair (Clark et al., 1994; Kumar et al., 2002; Gomes et al., 2007; Raghavan et al., 2010; Mashreghi et al., 2013). Although previous studies reported inconsistent results, with limited benefits in canine wounds (Falcão et al., 2002), the AH90 peptide accelerated reepithelialization and granulation tissue formation in mice (Liu et al., 2014). Likewise, our previous study found no significant benefits of frog skin in equine wound healing (Campebell et al., 2025). In contrast, the present study demonstrated significant improvements, suggesting that biomaterial processing may be critical to enhancing the therapeutic efficacy of frog skin in horses.

In this study, gamma irradiation effectively sterilized both frog and tilapia skin, as evidenced by the absence of bacterial and fungal growth after 72 h of culture. In contrast, frozen tilapia skin retained viable microorganisms despite prior chlorhexidine disinfection, indicating that freezing alone was insufficient to eliminate the resident microbiota. Morphological staining identified Gram-positive cocci and Gram-positive bacilli (such as *Enterococcus* spp. and *Streptococcus* spp.) and Gram-negative bacilli (such as *Pseudomonas* spp. and *Aeromonas* spp.), which were consistent with the resident bacterial microbiota described on the skin of Nile tilapia (Lima Junior et al., 2016). These findings demonstrate that sterilization is a critical prerequisite for the safe clinical use of fish skin xenografts and suggest that preservation by freezing alone may compromise biosafety. Biomaterial processing significantly influenced healing outcomes. Tilapia skin intended for use in occlusive dressings requires rigorous disinfection and sterilization, ideally accompanied by microbiological testing. Chemical sterilization and gamma irradiation are considered the most effective approaches (Alves et al., 2015).

The microbiological findings were consistent with the clinical outcomes observed in the frozen tilapia skin group. Compared with the other treatment groups, wounds treated with frozen tilapia skin (FTS) showed a higher frequency of contamination and excessive granulation tissue formation. Although causality cannot be established from the present study, these observations suggest that residual microbial contamination (Lima Junior et al., 2016) may have contributed to a prolonged inflammatory response and impaired tissue repair. This interpretation is supported by previous studies reporting successful clinical outcomes with glycerolized or chemically sterilized tilapia skin, in which microbial contamination was not observed (Barreira et al., 2022; Raymundo et al., 2025).

During the first postoperative week, all treatment groups exhibited the expected inflammatory response characterized by edema, hemorrhage, hyperemia, and clot formation, with the greatest intensity observed on day 3 and progressive resolution thereafter (Gomez et al., 2004; Resende et al., 2024; Campebell et al., 2025). However, wounds treated with sterilized frog skin (SFS) exhibited a moist wound environment and a transient yellowish exudate during the early healing phase. Rather than indicating treatment failure, this response may reflect maintenance of a moist wound environment, which is known to support cell migration, angiogenesis, and granulation tissue formation (Martins et al., 2003). In humans, wounds treated with frog skin as a xenograft did not show exudate accumulation (Adorno, 2005). The progressive reduction in exudate as healing advanced suggests that the dressing maintained favorable wound conditions without interfering with the normal progression of tissue repair.

Wound area reduction and contraction are key indicators of healing progression because they reflect the coordinated restoration of tissue architecture (Paiva et al., 2002). In the present study, wounds treated with frozen tilapia skin (FTS) had the largest residual wound areas in the early healing period and the lowest contraction rates throughout the study, indicating delayed wound repair. These findings were accompanied by persistent edema and exudation, suggesting prolonged inflammation that may have impaired the transition to the proliferative phase and delayed tissue organization. Although the mechanisms were not directly investigated, the inferior performance of FTS may be related to alterations in biomaterial integrity caused by freezing, which have previously been reported to affect collagen structure and wound–biomaterial interactions (Abbade and Lastoria, 2006; Sibbald et al., 2011; Lima Junior et al., 2017).

In contrast, sterilized frog skin (SFS) and sterilized tilapia skin (STS) produced significantly better healing outcomes from day 14 onward than our research group using non-sterilized frog skin under similar experimental conditions (Campebell et al., 2025). Although SFS showed slightly lower contraction during the initial evaluation, this difference was likely attributable to the larger baseline wound area rather than to reduced therapeutic efficacy. Overall, both sterilized biomaterials likely supported a more rapid progression of healing than FTS, suggesting that biomaterial processing may be as important as biomaterial composition in determining therapeutic efficacy. These findings are consistent with previous studies showing that gamma irradiation preserves the structural and biological properties of collagen-based dressings (Alves et al., 2015; Alves et al., 2018; Costa et al., 2020). Frog skin contains a wide range of structural proteins, antimicrobial peptides, lipids, and other bioactive molecules that have been associated with antimicrobial activity, modulation of inflammation, and tissue regeneration (Kumar et al., 2002; Gomes et al., 2007; Mashreghi et al., 2013). The improved performance observed in the present study suggestes that preservation of these biological properties, together with effective sterilization, may enhance the wound-healing potential of frog and tilapia skin in horses.

Negative contraction rates observed during the first postoperative week, particularly in the control and FTS groups, likely reflected transient wound enlargement due to edema, early inflammatory response, delayed myofibroblast recruitment and differentiation, a phenomenon previously described in equine wound healing (Theoret and Wilmink, 2013). This finding in the FTS group suggests that freezing compromised collagen integrity and reduced biomaterial interaction with injured tissue (Lima Junior et al., 2017). In contrast, both STS and SFS showed earlier wound-contraction recovery, indicating a more efficient transition from inflammation to tissue repair, due to gamma irradiation-preserved collagen structure and function (Costa et al., 2020) and regenerative potential (Bazaz et al., 2013). Collectively, these findings demonstrate that biomaterial processing influenced not only the magnitude of wound contraction but also the overall progression of healing, with sterilized biomaterials consistently outperforming frozen tilapia skin.

The clinical and histopathological findings consistently showed that wounds treated with sterilized frog skin (SFS) and sterilized tilapia skin (STS) progressed more rapidly through the inflammatory phase than those in the control group and the frozen tilapia skin (FTS) group. Hemorrhage resolved more rapidly in wounds treated with sterilized frog skin (SFS) and sterilized tilapia skin (STS) than in the control and frozen tilapia skin (FTS) groups. In contrast, hemorrhage persisted through day 14 in untreated wounds and those treated with FTS, indicating delayed stabilization of the wound environment. This pattern, together with the prolonged edema observed in FTS, supports the overall finding that frozen tilapia skin delayed progression through the early stages of healing (Lima Junior et al., 2017; Costa et al., 2020). Although the underlying mechanisms were not investigated, previous studies suggest that biomaterial preservation and bioactive compounds may influence hemostatic and inflammatory responses through hemostatic and anti-inflammatory peptides (Bazaz et al., 2013; Li et al., 2023) and by preserving biomaterial integrity after gamma irradiation (Alves et al., 2015).

The FTS group maintained elevated polymorphonuclear leukocyte infiltration throughout the study, indicating sustained acute inflammation, whereas both SFS and STS promoted earlier resolution of this response. STS consistently exhibited the lowest PMN counts, while SFS showed a marked reduction by day 7. These findings suggest that both sterilized biomaterials facilitated a more balanced inflammatory response than frozen tilapia skin, likely due to bioactive peptides with anti-inflammatory effects (Mashreghi et al., 2013; Raghavan et al., 2010). STS maintained the lowest PMN levels and a more balanced inflammatory profile through day 28, which are consistent with the more favorable macroscopic healing observed in these groups and may reflect preservation of the biological properties of the sterilized biomaterials.

Differences among treatments also became evident in the later stages of repair. Persistent histiocytic infiltration in the FTS group on days 21 and 28 suggested delayed progression toward tissue remodeling, whereas SFS and STS showed earlier, more organized macrophage recruitment. Because macrophages coordinate the transition from inflammation to tissue repair (Balbino et al., 2005; Reinke and Sorg, 2012), these findings indicate more efficient progression through the proliferative and remodeling phases in wounds treated with sterilized biomaterials. Although the molecular mechanisms remain to be determined, previous studies have shown that amphibian-derived peptides such as OA-RD17 and preserved collagen matrices may modulate macrophage activity (Li et al., 2023), thereby supporting tissue regeneration.

The more advanced tissue organization observed in the STS and SFS groups was reflected in earlier reepithelialization, particularly in STS-treated wounds, indicating faster restoration of the wound surface. In contrast, delayed epithelial coverage in the FTS paralleled the prolonged inflammatory response and reduced fibroplastic activity observed in this group. Collectively, these results suggest that biomaterial processing substantially influenced healing outcomes. Sterilized frog skin and sterilized tilapia skin may have promoted more efficient progression through the inflammatory, proliferative, and remodeling phases than frozen tilapia skin, supporting their potential as biological dressings for equine wound management.

As with other histopathological findings, sterilized frog skin (SFS) and sterilized tilapia skin (STS) likely promoted a more coordinated proliferative phase than frozen tilapia skin (FTS). Although neovascularization increased in all groups after day 7, angiogenesis was more organized and sustained in the SFS and STS groups, whereas the FTS group exhibited a transient angiogenic peak on day 14, accompanied by persistent inflammation. This pattern suggests that the increased vascular response observed in FTS may have reflected delayed tissue repair rather than effective progression through the proliferative phase. Our findings suggest that preserved collagen structure and bioactive compounds stimulate vascularization (Bazaz et al., 2013; Li et al., 2023).

Similar differences were observed for fibroplasia. Both SFS and STS promoted greater fibroblast proliferation and extracellular matrix deposition than FTS, indicating more advanced tissue organization. In contrast, the limited fibroplastic response in FTS paralleled its prolonged inflammatory profile, supporting the overall finding that freezing adversely affected the biological performance of the biomaterial. Although the mechanisms were not directly investigated, biomaterials likely enhance fibroblast proliferation and collagen synthesis (Liu et al., 2014; Assis et al., 2025), and increase hydroxyproline deposition and dermal organization (Sai et al., 1995; Raghavan et al., 2010).

Reepithelialization became evident after day 14 and occurred earlier and more extensively in STS-treated wounds, indicating faster restoration of the epithelial barrier. Together with the enhanced fibroplasia and organized angiogenesis observed in this group, these findings suggest that STS created a more favorable environment for tissue repair, owing to the beneficial effects of fatty acids and moist wound conditions (Garros et al., 2006). SFS produced a comparable regenerative response, although epithelial coverage progressed more gradually. Overall, the results indicate that biomaterial processing substantially influenced healing, with sterilized biomaterials consistently promoting more effective progression through the proliferative phase than frozen tilapia skin.

Infrared thermography complemented the clinical and histopathological evaluations by providing a noninvasive assessment of healing progression (Tunley and Henson, 2004; Redaelli et al., 2014). Thermal patterns closely paralleled the inflammatory responses observed in each treatment group. The FTS group exhibited prolonged temperature elevations, consistent with its persistent inflammation and delayed tissue repair, whereas wounds treated with sterilized frog skin (SFS) and sterilized tilapia skin (STS) showed earlier thermal normalization, suggesting more rapid resolution of the inflammatory phase. Hyperemia and cytokine-mediated vasodilation (Balbino et al., 2005), as well as elevated temperatures at wound edges, were associated with increased fibroblast and keratinocyte activity during granulation tissue formation (Reinke and Sorg, 2012). Notably, STS maintained moderate thermal activity through day 28, coinciding with the sustained angiogenesis and tissue remodeling (Costa et al., 2020) observed histologically. These findings may demonstrate that infrared thermography reliably reflects the temporal progression of wound healing and may serve as a useful adjunct for monitoring tissue repair in horses.

Overall, the results showed that biomaterial processing markedly influenced healing outcomes. Sterilized frog skin and sterilized tilapia skin may have promoted faster resolution of inflammation and a more coordinated progression through the proliferative and remodeling phases than frozen tilapia skin did. Although both sterilized biomaterials improved wound healing, distinct healing profiles were observed. Sterilized frog skin was likely associated with earlier modulation of inflammation and enhanced fibroplasia, whereas sterilized tilapia skin was associated with more consistent wound contraction and earlier reepithelialization. These complementary responses highlight the therapeutic potential of both biomaterials as biological dressings for equine wound management.

This study has some limitations. Although the controlled experimental design allowed direct comparison among treatments, the sample size was relatively small, and the wounds were experimentally induced under standardized conditions, which may not fully represent naturally occurring clinical wounds. In addition, molecular analyses of inflammatory mediators and growth factors were not performed. Future studies should investigate the biological mechanisms underlying the observed responses, optimize biomaterial processing protocols, and evaluate these dressings in clinical cases to further establish their therapeutic potential.

## 5 Conclusion

The high incidence of wounds in horses and the challenges of their repair underscore the need for effective biological dressings to improve healing outcomes. In this study, biomaterial processing emerged as a key determinant of therapeutic performance. Sterilized tilapia and frog skin consistently outperformed frozen tilapia skin, yielding more favorable clinical, histopathological, and thermographic healing profiles. Although both sterilized biomaterials enhanced wound repair, distinct healing patterns were observed: sterilized frog skin was associated with earlier resolution of the inflammatory phase, whereas sterilized tilapia skin produced more consistent wound contraction and reepithelialization. In contrast, frozen tilapia skin performed inferiorly, with delayed healing and greater evidence of inflammation. These findings support the potential of sterilized tilapia and frog skin as biological dressings for equine wound management and emphasize the importance of appropriate biomaterial processing to maximize therapeutic efficacy. Further studies involving larger populations, naturally occurring wounds, and molecular analyses are needed to validate these findings, elucidate the mechanisms underlying the observed responses, and establish their clinical applicability.

## Data Availability

The raw data supporting the conclusions of this article will be made available by the authors, without undue reservation.

## Supplementary material

SUPPLEMENTARY FIGURE S1 Preparation process for freezing tilapia skin: **(A)** Cleaning and removal of traces of muscle tissue, **(B)** Cleaning and immersion in 0.9% physiological saline solution, **(C)** Packaging in plastic bags for freezing. Preparation of the sterilized frog skin: **(D)** Cleaning and removal of traces of muscle tissue of frog skins, **(E)** Metal plates cleaned with water and neutral soap received a layer of frog skin oil to provide the necessary skin’s hydration, **(F)** The plates were exposed to a temperature of 28°C for 45 minutes to allow the skins to detach from the plates, **(G**) Frog skin packaged in surgical-grade paper which was sealed and sent for sterilization with Co-60 gamma rays.

SUPPLEMENTARY FIGURE S2 Performing skin wounds in horses: **(A)** Antisepsis of the area, **(B)** Marking the templates, **(C)** Incision in the shape of a 3 cm square and wounds performed, **(D)** One back side was used for clinical wound assessment, and the other was used to perform biopsies for microscopic evaluation, **(E)** Distribution of the experimental groups into their respective positions: from left to right sterilized frog skin (SFS), frozen tilapia skin (FTS), sterilized tilapia skin (STS) and control wound (CW). Thermographic evaluation in: **(F)** inside the wound, **(G)** around the wound and **(H)** at the wound edge.
